# The home field advantage of modern plant breeding

**DOI:** 10.1371/journal.pone.0227079

**Published:** 2019-12-26

**Authors:** Patrick M. Ewing, Bryan C. Runck, Thomas Y. J. Kono, Michael B. Kantar

**Affiliations:** 1 Department of Crop, Soil, and Microbial Sciences, Michigan State University, East Lansing, MI, United States of America; 2 GEMS Agroinformatics Initiative, University of Minnesota, Minneapolis, MN, United States of America; 3 Minnesota Supercomputing Institute, Minneapolis, MN, United States of America; 4 Department of Tropical Plant and Soil Science, University of Hawaii at Manoa, Honolulu, HI, United States of America; ICAR-Indian Institute of Agricultural Biotechnology, INDIA

## Abstract

Since the mid-20^th^ century, crop breeding has driven unprecedented yield gains. Breeders generally select for broadly- and reliably-performing varieties that display little genotype-by-environment interaction (GxE). In contrast, ecological theory predicts that across environments that vary spatially or temporally, the most productive population will be a mixture of narrowly adapted specialists. We quantified patterns of broad and narrow adaptation in modern, commercial maize (Zea mays L.) hybrids planted across 216 site-years, from 1999–2018, for the University of Illinois yield trials. We found that location was the dominant source of yield variation (44.5%), and yearly weather was the smallest (1.7%), which suggested a benefit for reliable performance in narrow biophysical environments. Varieties displayed a large “home field advantage” when growing in the location of best performance relative to other varieties. Home field advantage accounted for 19% of GxE and provided a yield increase of 1.01 ± 0.04 Mg ∙ ha-1 (7.6% relative to mean yield), yet was both smaller than predicted by a null model and unchanged across time. This counterfactual suggests that commercial breeding programs have missed an opportunity to further increase yields by leveraging local adaptation. Public breeding programs may pursue this opportunity by releasing specialist varieties that perform reliably in narrow environments. As seed sources are increasingly privatized and consolidated, this alternate strategy may compliment private breeding to support global food security.

## Introduction

The breeding of high-performing crop cultivars is fundamental to productive agricultural systems. Releasing these cultivars usually includes evaluating cultivar performance across a variety of environments to select broadly reliable performers [1,2]. Evolutionary biologists have long recognized that individual organisms in natural systems tend to perform better in their location of origin [3,4]. This ability of individuals to adapt to local conditions determines population-wide fitness: when environmental heterogeneity across a region is large, the most fit population is predicted to be a mixture of locally-adapted specialists [5]. Leveraging this ecological theory can provide insights into how breeders might adapt crops under climate instability, which amplifies spatio-temporal environmental heterogeneity [6]. In particular, we consider the extent to which a maximally-productive, regional crop population should be a mixture of specialists or generalists, and how this information may guide breeding strategies to meet projected global food demands [7,8].

The regional composition of a crop’s population is restricted by the availability of cultivars [8]. This contrasts with natural systems, where population composition is dictated by the reproductive success of individuals over generations [3,9]. Local cultivar availability results from the artificial selection strategies of breeding programs. Historically, selection has occurred at relatively narrow spatial scales and environments, resulting in a regionally heterogeneous mixture of locally-adapted landraces (traditional heirloom cultivars) [10]. Previous research on landrace fitness–defined in terms of seed yield, size, or other similarly anthropocentric attributes–has shown that landraces tend to have stable fitness in their “home” environment, but perform poorly in other environments [11]. For example, maize landraces in Mexico have clear local adaptation along altitudinal gradients, and the most fit maize population across altitudes was a mixture of landraces [12,13].

Modern breeding programs have taken an alternative approach, which has helped ensure that global food production could keep pace with caloric demand [2, 14]. One common strategy, which contrasts with landraces, is expanding the size of a variety’s home environment by attempting to find varieties that perform well reliably across distant locations [1]. For example, maize varieties in the United States might be released at the state level [1]. In contrast to ecological theory, breeding theory encourages this expansion by emphasizing the use of parents with stable phenotypes—i.e. display a minimal genotype-by-environment interaction—to develop broadly-performing candidate lines [1]. This is a practical approach that historically minimized costs by maximizing the expected performance of a single variety across a large area.

Despite past success, given increasing climate instability that is amplifying field variability, breeders may need new approaches to maintain crop improvement [6]. Ecological theory suggests that the practice of planting single varieties adapted to large regions may not maximize regional agronomic production; instead, as environmental heterogeneity increases either spatially or temporally, the most productive crop population is more likely to be a mixture of narrowly-adapted varieties [5]. This is because the spatial and climatic extent of a variety’s range affects overall fitness, with sub-populations established in relatively incompatible “fringe” conditions producing fewer offspring [5,15]. This is recognized in the agronomic principle of matching management, including variety selection, to local environmental conditions, even if this management partially obviates environmental heterogeneity [8, 16]. Therefore, quantifying local adaptation in crop populations has two practical implications: informing the selection and planting of appropriate varieties at the field scale; and informing the development of breeding strategies to improve regional crop fitness across localized agronomic settings [5,17,18].

To quantify local adaptation in natural populations, studies often use common gardens and reciprocal transplants across geographically separate regions [19]. Regional agronomic trials provide a robust common garden experiment because a large number of genetically identical varieties are replicated across locations with different environmental conditions. We used twenty years of maize hybrid yield trail data from the University of Illinois to quantify the extent to which modern, commercial crop varieties are specialist, local performers or generalist, broad performers. We define local adaptation as the expected yield advantage conferred by growing in a variety’s home environment—the home-field advantage—where the home environment is the location where a variety performed best relative to other varieties. The focal time period, 1999–2018, corresponds to the adoption of new technologies, a greater number of field sites, and more efficient experimental designs, which have reduced the length and cost of breeding cycles [2, 20, 21]. We hypothesized these developments would also increase companies’ abilities to target narrower home environments, as evidenced by an increase in home-field advantage. The maize varieties in this dataset are modern, commercial, and broadly planted. Therefore, our results provide a unique window into the commercial breeding strategies that underlie the germplasm supporting maize production.

## Materials and methods

### Data acquisition

Maize yield data were retrieved from the University of Illinois Corn Hybrid Variety Trials conducted from 1999 to 2018 (http://vt.cropsci.illinois.edu/corn.html and S1 File). Illinois is 628 km north-south and 338 km east-west; trial sites are grouped into four regions, north, east, west, and south. Each region contains three sites; each site contains three replicates. Each year, companies voluntarily enter a number of varieties for at least one of the four regions. Not all sites were represented in every year; the dataset we used here contains 14 sites that were represented in more than three years. The mean yield of three replicates grown at each variety-site-year combination is reported. Because varieties are rarely entered in more than one region or across more than three years, the analysis focused on ‘check’ varieties, which researchers planted as controls at each location. Data from 2012 and 2016 were either not available or had only two check varieties, and so were excluded. This resulted in a total of 230 uniquely labeled varieties, which we assume corresponded to independent genotypes.

Site environmental conditions were also collected (S1 Table and S2 File). The latitude and longitude coordinates of each trial site were used to query the WorldClim database for nineteen environmental variables at a resolution of 30 arc-seconds (approximately 1 km; see http://www.worldclim.org/). The bioclimatic variables represent annual trends between 1960–1990, and consist of seasonality and extreme or limiting environmental factors that are often used in ecological niche modeling [22]. The sampling locations were also used to query the ISRIC database (World Soil Information database) for seventeen biophysical variables averaged across depth classes (see http://www.isric.org/) [23]. Site ordination is available in S1 Fig.

### Analysis

Statistics were performed in base R v3.4.4 except as noted [24], and plots made using ggplot2 v2.2.1 [25]. Centering and scaling refer to a group mean of zero and standard deviation of one, respectively.

The first task to understand local adaptation was characterizing environmental heterogeneity across locations. The University of Illinois Extension Service categorizes field sites based on geographic location into north, east, west, and south regions. In contrast, we used two empirical approaches to derive regions with similar conditions. In a bottom-up approach, site-year weather and soil data were centered, scaled, and then ordinated using principal component analysis using the prcomp function in base R. In a top-down approach, average site yields by year were ordinated using a Bayesian latent variable approach using the bcfa function in the package blavaan v0.3.2 [26,27]. Results are analogous to non-metric multidimensional scaling; this approach was used due to incomplete representation of sites across years. Data were centered and scaled within year before analysis, which removed temporal effects on yield (See S1 Fig for details).

We estimated overall home field advantage as the additional yield benefit conferred to a variety after accounting for inherent site and genetic yield potential [28]. The first step was to assign each variety a “home” site, the site where a variety performed best relative to other varieties. We calculated relative yields within each site-year by centering and scaling. The home site for each variety was the site with the maximum average relative yield across years. We then modeled observed yield as:

Yieldijk=genotypei+sitej+yeark+sitej*yeark+is_homeij+ε Eq 1

Where *is_home* is an indicator for whether site *j* is the home site for variety *i*; its coefficient is the home field advantage. Coefficients on variety, site, and year respectively represent the genetic potential of a variety, the site’s productive potential, and the year’s weather suitability. Variety-year, variety-site, and 3-way interactions were not estimable due to low replication of check varieties across years and already-aggregated variety performance data at each site. We compared this full model to a control model that did not include a home site term using Akaike and Bayesian information criteria (AIC, BIC). Finally, we investigated the proportion of variation in yield explained by each factor of the above model using a Type-II analysis of variance using the Anova function in car v2.1–4 [29].

We estimated the trajectory of home field advantage across years. Each year, we estimated a home field advantage found in each year using Eq 1, but without terms including year. We ran this model using median quantile regression using the rq function in quantreg v5.33 [30]. Quantile regression is robust to non-normal response distributions, which were observed in some years; results were broadly similar to ordinary least squares regression. The coefficient for the *is_home*_*ij*_ term, the yearly home field advantage, was then regressed against year.

Finally, we compared our estimates of home field advantage to what we might expect given a neutral breeding approach—i.e., one that did not favor either broad or narrow performance—using permutation. Within each site-year combination, we randomly reassigned yields to different check genotypes. This approach preserves within-site-year variation, but negates genotype advantages. Based on this permuted data, we then re-assigned a home site and calculated home advantage for each year as described above. We repeated this 999 times.

## Results

### Similarity among environments

A critical consideration in any analysis of local adaptation is the similarity of candidate environments. We approached this issue using both bottom-up (i.e. biophysical and bioclimatic variables) as well as a top-down (i.e. yield) approaches. Each ordination approach resulted in the same two site-groups: a “southern” group, and the rest of Illinois (S1 Fig). Principal component (PC) analysis revealed extensive multicollinearity among environmental variables, with the first four PCs explaining ~88% of the total environmental variation (S1B Fig). The first two PCs largely recreated the geographical distribution of field sites. In contrast with the University of Illinois grouping the 19 sites into four regions, based on climatic and biophysical, regions are largely represented solely by a north to south gradient. Sites in northern Illinois were colder and drier (i.e., continental climate) compared to southern Illinois (S1C Fig). Confirming that this gradient defines the environment maize experienced, an ordination of sites based on mean maize yield by year revealed similar, north-south patterns (S1D Fig).

### Sources of yield variance and a home field advantage

**Fig 1:**
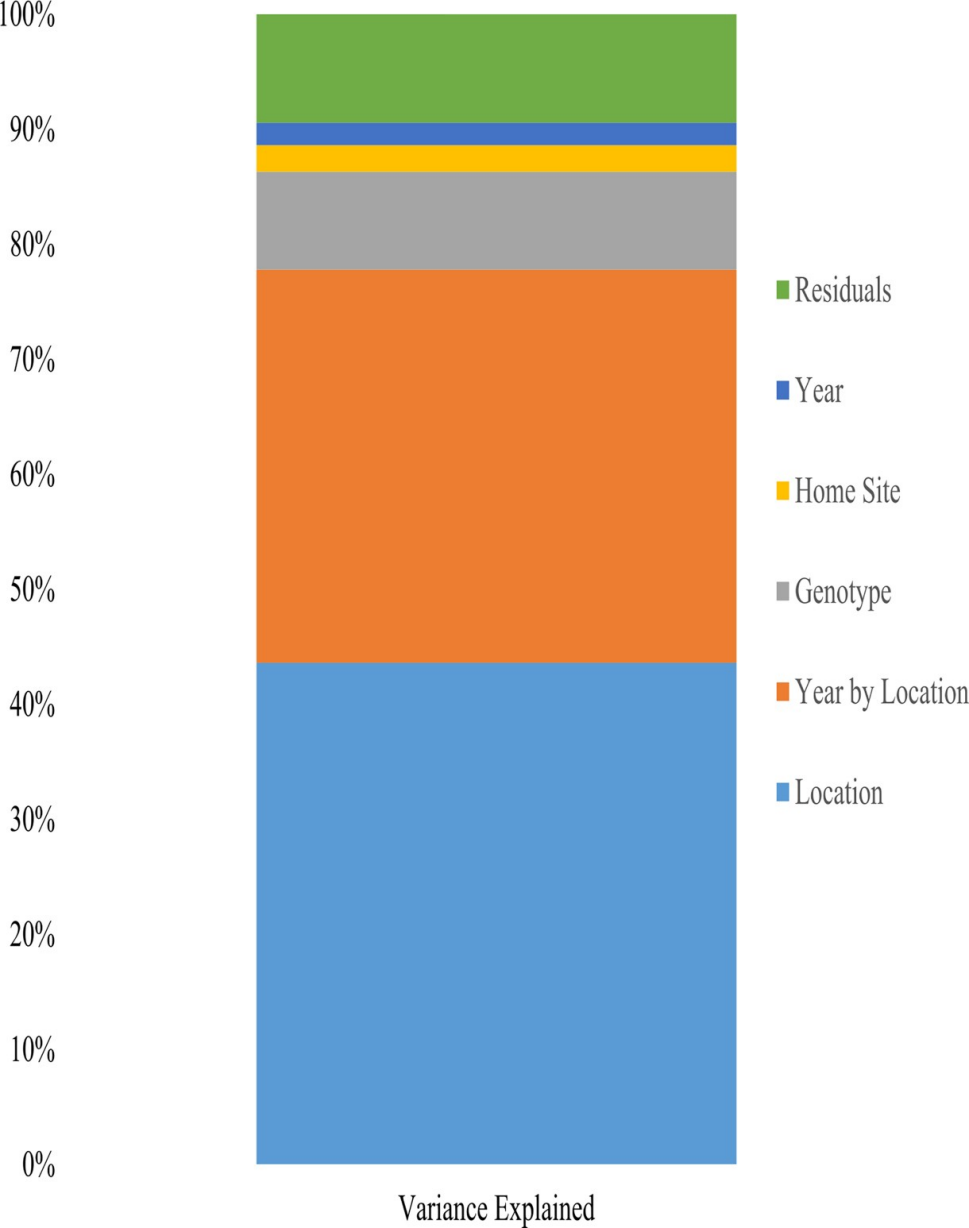
Sources of commercial maize yield variation across 20 years of yield trials. Variation sources were identified with an analysis of variance; all are significant at p < 0.001.

We further investigated yield and local adaptation by partitioning yield variation into genetic and environmental components (Fig 1). Breeding for broad performance across environments is often justified by the assumption that year-to-year variation is large, and so each site-year represents a unique environment. Results demonstrate year-on-year variation to be the smallest source of yield variation in Illinois (1.8%, p < 0.001). A site-year interaction had a larger effect, accounting for 33.6% of yield variation (p < 0.001); indeed, this interaction was more important than genotype variation (10.2%, p < 0.001). However, location alone was the single largest source of variability in maize yield across Illinois (42.7%, p < 0.001). This suggests that, given the environmental heterogeneity observed in Illinois (S1A Fig), breeding location-specialized maize varieties could be fruitful.

The above partitioning leaves 11.7% of maize yield variation unexplained. Formally, this residual variation is a combination of measurement error and genotype-by-environment effects. We found that incorporating a variable that flagged whether a site was a variety’s "home" site (see Methods) accounted for 19.7% of this residual variation, or 2.3% of overall maize yield variation (p < 0.001). Comparing models with and without a home site term (see Eq 1) underlined the importance of a variety growing in its home site (AIC: without = 6153, with = 5538; BIC: without = 8619; with = 8010). The importance of a home site term in predicting yield suggests habitat specialization in the germplasm studied. This specialization came at a trade-off with stability across environments, as varieties that showed high home advantages also had more variable yields across environments (R^2^ = 0.33, p < 0.001;S2 Fig).

**Fig 2:**
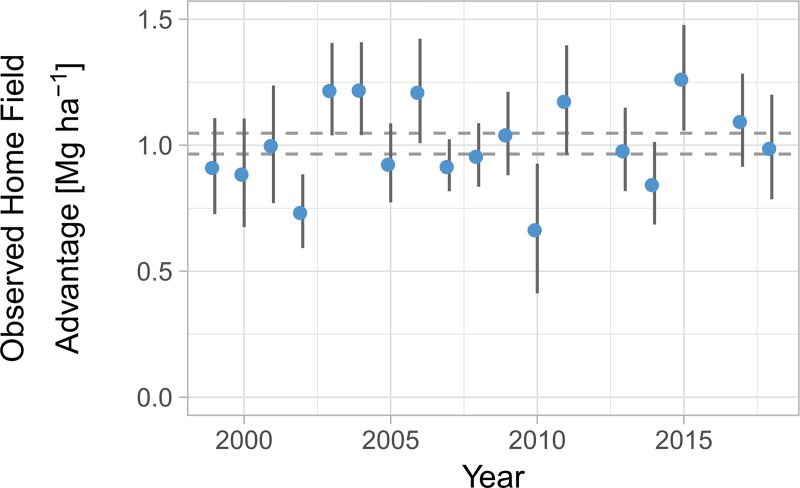
Home field advantage observed in commercial maize varieties in Illinois across time. Dashed horizontal lines represent the error around the overall mean. Errors are standard errors bootstrapped from median quantile regression.

**Fig 3:**
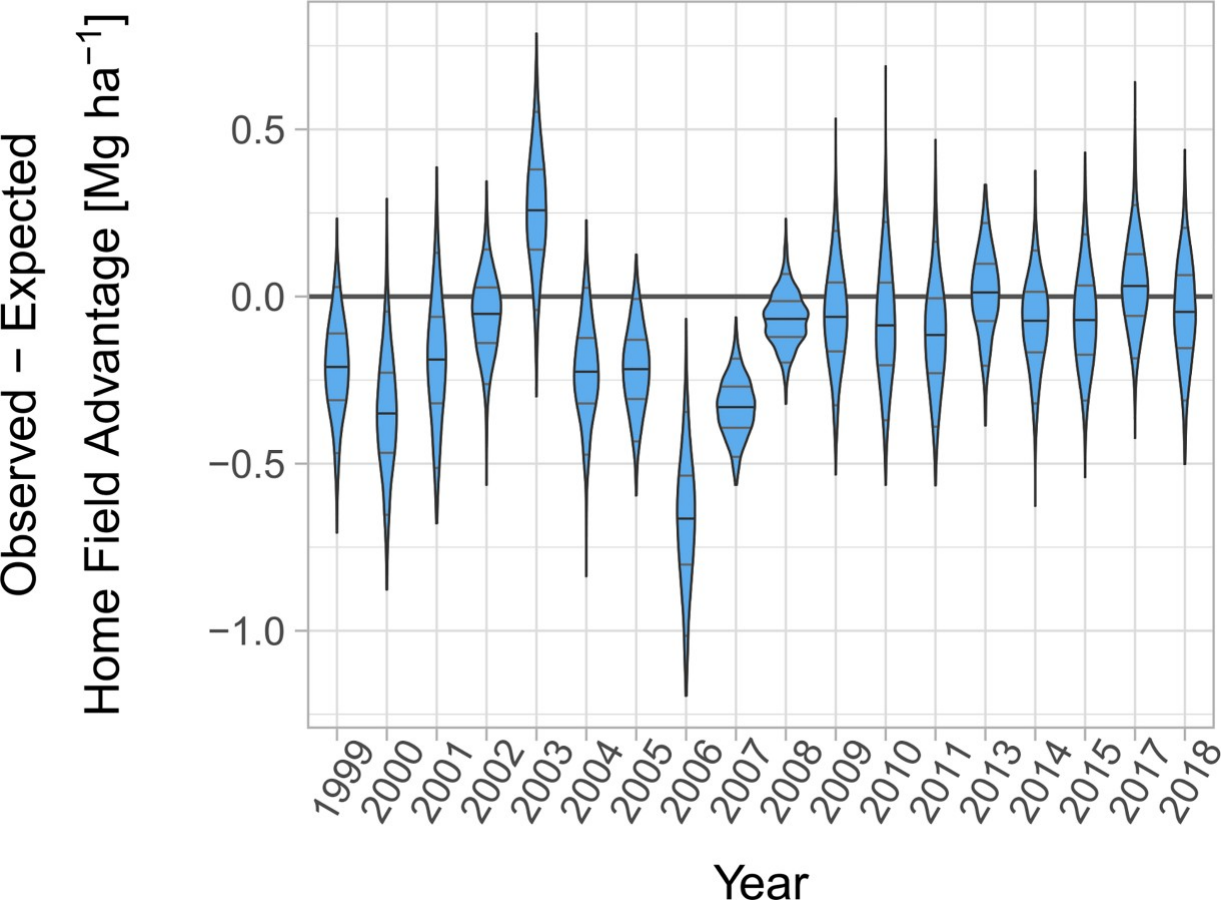
Permutation-based comparison of observed home field advantage (Fig 2) with that expected in the absence of a breeding strategy. Expected home field advantage was estimated by 999 permutations, where yield was reassigned to varieties within each site-year. Horizontal lines denote the 50th percentile (black), 50%, and 90% intervals (gray). Band width is proportional to the density of observations.

Overall, the home field advantage translated to an ‘unlocking’ of 1.01 ± 0.4 Mg ∙ ha^-1^ more than expected given site, year, and genetic potentials. This represents a yield increase of 7.6% over the dataset mean of 13.29 ± 0.4 Mg ∙ ha^-1^ (Fig 2). This agronomically significant yield advantage does not alone provide evidence of breeding for habitat specialization and may be an overestimate due to the Beavis effect, which states that effects tend to be overestimated when sample sizes are small and pre-screening selects likely candidates [31]. To contextualize this result and possible bias, we permuted the data to estimate home field advantage based purely on the variation within the dataset. Each permutation should be subject to a similar Beavis effect, if present, as the original data. Permutation revealed that this home advantage was consistently, and in some years significantly, smaller than expected in the absence of a breeding strategy (Fig 3). This smaller-than-expected home advantage suggests that, as predicted, breeding has emphasized broad performance at the expense of local specialization.

We had further hypothesized that, with advancements in breeding abilities, we would observe an increase in local adaptation across the 20 years this dataset spans. Our observations did not support our hypothesis, as a home field advantage showed no change across the study period (adj. R^2^ = 0.04, p = 0.20; Fig 2). This constant home advantage contrasted with a background of yearly maize yield increases that averaged 0.22 ± 0.3 Mg ∙ ha^-1^ yr^-1^ (adj. R2 = 0.82, p < 0.001; S3 Fig).

## Discussion

Food systems are increasingly globalized and incorporate a growing number of regionally-important crop species, even as food demand grows to meet the needs of a projected 10 billion people by 2050 [32,33]. Meanwhile, the private sector is supplanting public breeding programs and seed saving as the dominant source of seed for a larger number of crops and a larger number of countries, with unknown implications for regional and long-term food security [14]. These developments demand a greater scrutiny of established breeding strategies in order to release crops adapted to new and changing environments. We found that modern, commercial maize varieties display an agronomically significant amount of habitat specialization in the form of a 1.01 ± 0.4 Mg ∙ ha^-1^ home field advantage. This is a 7.6% yield increase, which equates to more than four years of observed year-on-year yield gains. Habitat specialization is the antithesis of the established breeding regime, which started in the early 1900s and has attempted to develop individual ‘good’ lines over wide geographic areas [34]. This success of this regime has been foundational to increases in global food security in the 20th century, and our null permutations suggest that yield gains may be larger if breeders can target habitat specialization [35].

While at odds with breeding theory, our results do agree with the expectation from natural systems that local adaptation is critical for an organism’s survival, even in species that have acquired broad ecological niches and in stochastic climates [5,36]. This expectation has been explored in crop landraces, which often display high levels of local ancestry and local specialization [37]. However, modern breeding approaches coincided with a homogenization of management practices as well as of genetics, which are assumed to reduce environmental variation [16]. Our results challenge this assumption. We found that, over the past 20 years, the largest source of maize yield variation in a single state, Illinois, was location (42.7%), followed by the location-year effects (33.6%). These are independent of the observed home field advantage. That we observed these results in a dryland region, where yearly weather-related variation is difficult to mitigate, highlights how site-specific varieties may be valuable to farmers.

The results of this study speak to the variety development strategies of private breeding companies, which released most of the varieties used in this study. We were particularly interested in how these strategies may have changed as companies adopted advances in genotyping, phenotyping, and statistics [20, 21]. We did not observe an increase in home field advantage did not increase across time. One interpretation, which we cannot rule out, is that close kinship within breeding programs reinforced varietal similarity due to inheriting diploid genotypes or to epistasis; this would limit the ability of our method to detect local adaptation at the individual variety level [38]. Our analysis assumed each variety was genetically independent. Within companies, subsidiaries, and/or dynamic commercial partnerships, this assumption does not hold. Nonetheless, genetic independence is a reasonable assumption given our immediate aim of investigating population-wide genotype-by-environment interactions, and a necessary assumption given the secrecy of private breeding programs.

A more likely interpretation is that private breeding programs do not view local adaptation as an economically viable way to improve varieties. As we discuss below, a breeding program that emphasizes narrowly adapted varieties requires either more field sites in a smaller area, or a longer release cycle. Both could induce an economic competitive disadvantage, as a smaller area suggests a smaller market size, while companies may further feel pressure to maintain the short-term, steady yield increases we observed. Moreover, bringing in new genetic diversity, while often discussed in scientific literature, may require considerable risk and investment [39]. Combined, these pressures may favor a business-as-usual, short-release, broadly adapted breeding strategy. Whether this is a sound long-term strategy for farmers and food security is uncertain.

### Opportunities for local adaptation in breeding?

Breeding has benefited from a wealth of new tools that improve selection efficiency, including genomic selection, marker-assisted backcrossing, and data mining [21]. Our results, combined with the three-year release cycle for privately-bred maize varieties [40], suggest that private breeding programs have leveraged these tools release new and broadly adapted varieties more quickly—i.e. to increase the temporal density of variety release. To generate enough site-year replicates to rapidly identify genetic markers that consistently increase fitness, companies establish a large number of field sites within a broad region [40]. The resulting broadly adapted selection strategy may not reflect the best approach to achieving the highest regional yields. With growing weather instability with climate change, whether a few weather-years can be considered ‘representative’ is uncertain [6]. Moreover, predictions of population composition across heterogeneous environments suggest that generalism may not be optimal for overall productivity [5]: multiple varieties with high local performance may outperform a single, generalist variety at the regional scale. Our results highlight this unrealized potential.

Our results suggest potentially large, regional yield benefits from planting locally adapted varieties. Developing these locally adapted varieties may be a fruitful strategy for public breeding programs, which generally do not have the resources to support such a large number of field sites in a given year. Such a strategy would produce varieties that resemble landraces—narrowly adapted, yet highly reliable across years. A critical distinction is that “local” may now be defined by biophysical proximity, thanks to the ability to collect high-resolution spatio-temporal datasets that support envirotyping, to complement advances in plant phenomics and genomics [41]. With this data, the parameters that define the environmental suitability of a specific genotype may be identified and used for artificial selection by breeders as well as for variety placement on-farm. Some of these factors are well-established: season length, soil nutrients, and expected rainfall distributions. Other niche parameters are just emerging in importance, such as microbiomes, which vary consistently with bioclimatic and soil conditions, and moreover display compatibility with different crop genotypes [42,43]. Directing ecological theory to identify agroecological niches that correspond to peak variety performance is essential for spatially dense public breeding programs to develop.

This approach is reminiscent of Additive Main effects and Multiplicative Interaction (AMMI) models, which define “mega-environments” of co-varying yield, and then describe them using ordination of environmental variables [2]. Our northern and southern derived regions correspond to mega-environments, although we also observed significant variation within these mega-environments. AMMI models are commonly used to increase statistical power and/or design experiments that uncover genetic effects more efficiently [44]. Our results suggest that commercial breeding programs use AMMI models for greater statistical power; if companies used AMMI to breed narrowly-adapted varieties, as advocated [44], we should have found an increase in home field advantage over our twenty-year study period. Rather than relying on a large number of disparate field sites to generate site-year replicates, public programs may use a smaller number of field sites across more years to generate narrowly-adapted varieties.

Such an approach comes with challenges, such as incorporating new genetic variation into breeding programs to better take advantage of these narrower niches. As environments become more heterogeneous, the prevalence of rare alleles is expected to increase in proportion to the (recent) frequency in which they are beneficial [5]. Agronomically significant heterogeneity occurs within fields, which can have markedly different soil properties [45]. Climate change-induced weather instability compounds this soil heterogeneity, further increasing the variety of environments crops experience [6]. While this article is not the first to call for increasing genetic diversity in crops [46], we provide a concrete and potentially fruitful application for this genetic diversity: leveraging local compatibility.

The broad-adaptation breeding strategy observed here in maize is likely representative of other commodities, particularly those primarily bred in the private sector. Further analyses might study whether crops that have not been part of the major private sector breeding efforts also display suppressed local adaptation. Doing so may shed additional light on the extent to which public breeding programs have and could contribute to the sustainable increase in agricultural production by providing highly tuned varieties for precise placement on the landscape.

## Conclusion

Commercial breeding emphasizes rapid release cycles and broad adaptation, accelerated by advances in breeding technology, and which both benefit and rely upon economies of scale [14]. This spatial scale of commercial trials is difficult for publicly bred and minor crops to replicate; moreover, broadly adapted varieties may not benefit individual farmers’ bottom lines. The combination of a large home field advantage, plus evidence that this home field advantage has not been the focus of breeding program improvement, suggests that commercial breeding has sacrificed yield to streamline variety release.

This implies an opportunity to improve food security and crop quality. The pillars of sustainable intensification—increasing agricultural output while decreasing environmental and social costs—include matching management to environmental conditions [8, 47]. Variety selection is a crucial management choice [48]. Breeding programs, therefore, have a considerable opportunity to further sustainable intensification by taking advantage of the locally adaptive potential of varieties. Public breeding programs are well suited to lead this pursuit, which the convergence of phenomics, envirotyping, and genotyping can support. While pursuing locally adapted varieties may require reevaluating breeding objectives and strategies, this reevaluation a worthwhile step toward improving sustainability and food security.

## Supporting information

S1 Fig Site yield comparisons a) Locations of Illinois yield trial fields. b) Ordination of weather and soil data for each site. c) bioclimatic variables loadings on to axes in b. d) Latent variable ordination of yield responses.
(PDF)

S2 Fig Home advantage vs variation across environments. (PDF)

S3 Fig Yield increases across time. (PDF)

S1 File Yield data used in this study. (CSV)

S2 File Climate and soil data used in this study. (CSV)

S1 Table Bioclimatic and biophysical variables used to place sites in environmental space. (DOCX)
