# Supplementary material for: The home field advantage of modern plant breeding
Source: PLoS One. 2019 Dec 26;14(12):e0227079. doi: 10.1371/journal.pone.0227079 (PMC6932805; doi:10.1371/journal.pone.0227079)
Supplement: S1 Fig — Site yield comparisons a) Locations of Illinois yield trial fields. b) Ordination of weather and soil data for each site. c) bioclimatic variables loadings on to axes in b. d) Latent variable ordination of yield responses. (PDF) [file pone.0227079.s001.pdf]

Supplementary Information

Supplementary Methods

To determine the extent to which sites displayed coordinated yield responses in line with regional labels, yield data were ordinated on two, orthogonal axes using a Bayesian latent variable approach. This is analogous to non-metric multidimensional scaling, and allows flexibility for missing data. Only sites represented in more than five years were used. Yields were centered and scaled within year to remove temporal variation. To ensure model identification and rotational determinacy, Goodfield was restricted to negative values on axis 1 and given a score of 0 on axis 2, while Monmouth was restricted to negative values on axis 2 (Merkle and Wang, 2018). These choices affect axis orientations and scales, but not inferences. Cauchy (0, 2.5) priors were used for loadings to aid convergence (Gelman and Hill, 2006). Analysis was performed using the bcfa() function from the *R* package, blavaan (Merkle and Rosseel, 2016).

Figure S1: Site yield comparisons
a) Locations of Illinois yield trial fields.

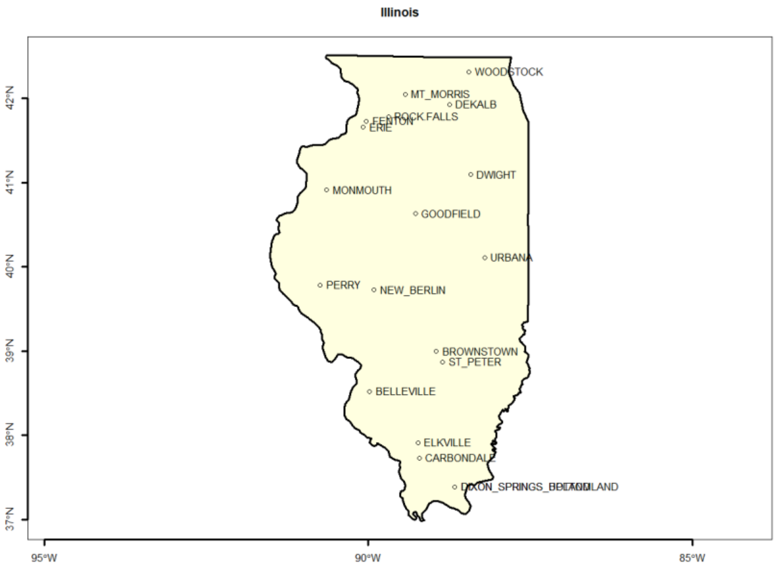

b) Ordination of weather and soil data for each site.

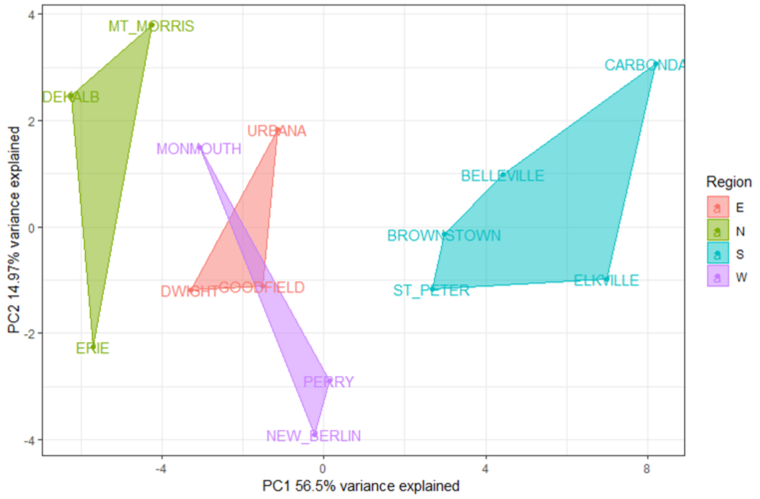

c) bioclimatic variables loadings on to axes in b).

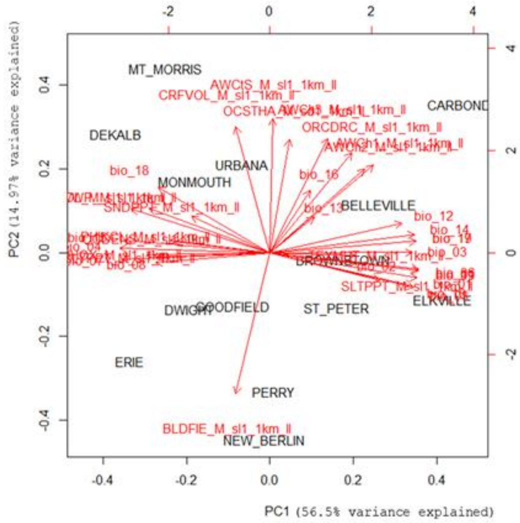

d) Latent variable ordination of yield responses

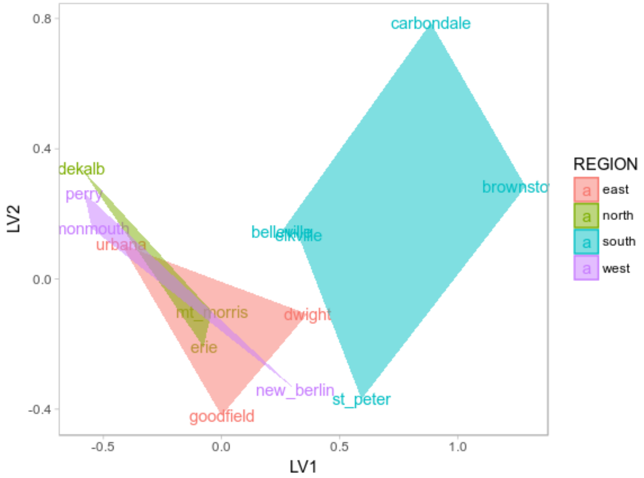

a) Locations in Illinois where sites data were collected. b) Ordination of field sites based on bioclimatic variables. c) bioclimatic variables loadings on to axes in a). In b) and c), groupings are based on regions as defined in the original dataset. d) ordination of field sites based on yields. Sites are restricted to those represented >5 years, constrained by year. Goodfield and Monmouth were restricted to enable model specification (Supplementary Methods). Latent variable labels (LV1 and LV2) are arbitrary.
