# Supplementary material for: The home field advantage of modern plant breeding
Source: PLoS One. 2019 Dec 26;14(12):e0227079. doi: 10.1371/journal.pone.0227079 (PMC6932805; doi:10.1371/journal.pone.0227079)
Supplement: S2 Fig — (PDF) [file pone.0227079.s002.pdf]

Figure S2: Home Advantage vs Variation Across Environments

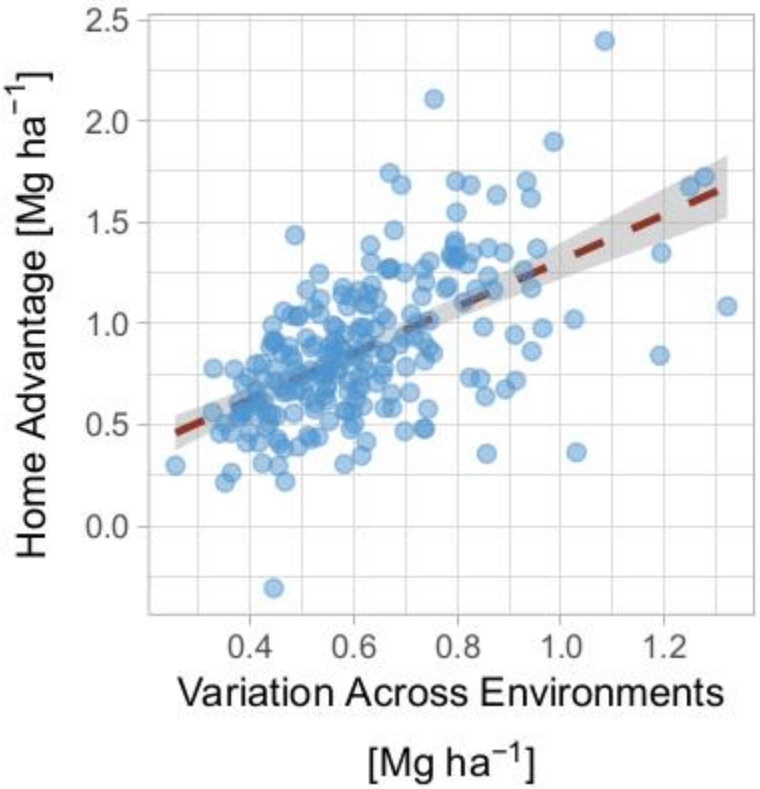


```
Coefficients:
               Estimate Std. Error t value Pr(>|t|)
(Intercept)     0.16797    0.06992   2.402   0.0171 *
sqrt(VARIANCE)  1.14129    0.10800  10.567   <2e-16 ***
---
Signif. codes:  0 '***' 0.001 '**' 0.01 '*' 0.05 '.' 0.1 ' ' 1

Residual standard error: 0.303 on 223 degrees of freedom
Multiple R-squared:  0.3337,          Adjusted R-squared:  0.3307
F-statistic: 111.7 on 1 and 223 DF,  p-value: < 2.2e-16
```
