# Supplementary material for: The home field advantage of modern plant breeding
Source: PLoS One. 2019 Dec 26;14(12):e0227079. doi: 10.1371/journal.pone.0227079 (PMC6932805; doi:10.1371/journal.pone.0227079)
Supplement: S3 Fig — (PDF) [file pone.0227079.s003.pdf]

Figure S3: Yield increases across time.

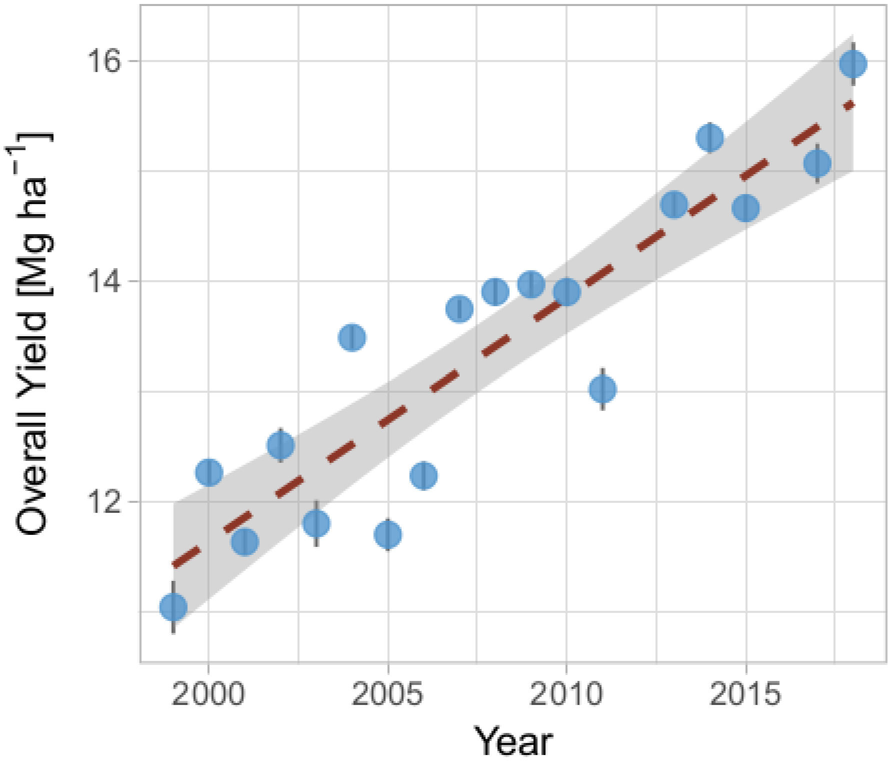

Errors are standard errors. $R^2 = 0.54$.

```
Coefficients:
             Estimate Std. Error t value Pr(>|t|)
(Intercept) -430.55276   50.65005  -8.501 2.50e-07 ***
YEAR           0.22109    0.02523   8.765 1.66e-07 ***
---
Signif. codes:  0 ‘***’ 0.001 ‘**’ 0.01 ‘*’ 0.05 ‘.’ 0.1 ‘ ’ 1

Residual standard error: 0.6126 on 16 degrees of freedom
Multiple R-squared:  0.8276,  Adjusted R-squared:  0.8169
F-statistic: 76.82 on 1 and 16 DF,  p-value: 1.664e-07
```
