# Supplementary figures and images for: The home field advantage of modern plant breeding
Source: PLoS One. 2019 Dec 26;14(12):e0227079. doi: 10.1371/journal.pone.0227079 (PMC6932805; doi:10.1371/journal.pone.0227079)

## **Table S1. Bioclimatic and biophysical variables used to place sites in environmental space.**

**
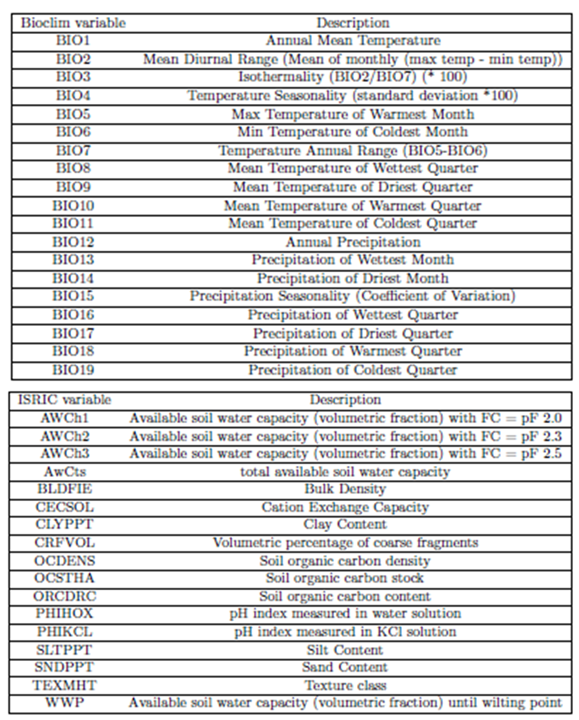
**

Supplement: S1 Table — (DOCX) [file pone.0227079.s006.docx]
